# Genetic Variants of *CITED2* Gene Promoter in Human Atrial Septal Defects: Case-Control Study and Cellular Functional Verification

**DOI:** 10.3390/jcdd9100321

**Published:** 2022-09-23

**Authors:** Zhuo Chen, Huan-Xin Chen, Hai-Tao Hou, Xiu-Yun Yin, Qin Yang, Jun Han, Guo-Wei He

**Affiliations:** 1 School of Pharmacy, Drug Research & Development Center, Wannan Medical College, Wuhu, Anhui 241002, China and The Institute of Cardiovascular Diseases, TEDA International Cardiovascular Hospital, Tianjin University & Chinese Academy of Medical Sciences, Tianjin 300457, China; 2 The Institute of Cardiovascular Diseases and Department Cardiovascular Surgery, TEDA International Cardiovascular Hospital, Tianjin University and Chinese Academy of Medical Sciences, Tianjin 300457, China; 3 School of Pharmacy, Drug Research & Development Center, Wannan Medical College, Wuhu, Anhui 241002, China

**Keywords:** atrial septal defect, CITED2, genetic, cardiac, heart

## Abstract

Atrial septal defect (ASD) is one of the most common forms of congenital heart disease (CHD). Genetic variants in the coding region of the CITED2 gene are known to be significantly correlated with CHD, but the role of variants in the promoter region of CITED2 is unknown. We investigated variants in the promoter of the CITED2 gene in 625 subjects (332 ASD and 293 healthy controls) through Sanger sequencing. Four variants in the CITED2 gene promoter were found only in eight ASD patients with zero occurrence in the control subjects (one case of g.4078A>C(rs1165649373), one case of g.4240C>A(rs1235857801), four cases of g.4935C>T(rs111470468), two cases of g.5027C>T(rs112831934)). Cellular functional analysis showed that these four variants significantly changed the transcriptional activity of the CITED2 gene promoter in HEK-293 and HL-1 cells. Electrophoretic mobility change assay results and JASPAR database analysis demonstrated that these variants created or destroyed a series of possible transcription factor binding sites, resulting in changes in the expression of CITED2 protein. We conclude that the variants of CITED2 promoter in ASD patients affect the transcriptional activity and are likely involved in the occurrence and development of ASD. These findings provide new perspectives on the pathogenesis and potential therapeutic insights of ASD.

## 1. Introduction

Congenital heart disease (CHD) is one of the eight leading causes of infant death, accounting for 30% of fetal deaths, with a prevalence of nearly 1.8 in 100 live births [1]. Atrial septal defect (ASD) is a major subtype of CHD, accounting for 7–10% of CHD in children and 25–30% of CHD in adults [2,3]. The true incidence of ASD is often higher, probably due to early classification and diagnostic problems [4]. Although ASD is considered a simple defect, due to the huge heterogeneity in anatomy and time-related complications (mainly arrhythmias, thromboembolism, right heart failure, and pulmonary arterial hypertension in some patients), optimal diagnosis and treatment of ASD remain challenging [5]. Like other congenital heart diseases, the pathogenesis of ASD is still not clear, and it may be related to genetic and environmental factors [6]. Many epidemiological studies believe that genetic factor is the most important reason [7]. With the breakthrough of molecular genetic technology, point mutation, chromosomal aneuploidy and copy number variant are considered the main genetic causes of congenital heart disease [8].

CITED2 (OMIM: 602937) is a cAMP response element binding protein (CBP)/p300 interacting transcriptional regulator and a negative regulator of HIF1A, by competing with HIF1A for binding to CBP/p300 [9]. The transcriptional regulator CITED2 is required for normal embryonic development and is involved in the development of multiple organs. CITED2 knockout embryos exhibit cardiac malformations, adrenal hypoplasia, neural crest defects, and anencephaly [10].

Variants in the coding region of the CITED2 gene lead to a range of cardiac malformations and congenital heart defects, such as ventricular septal defect (VSD), ASD, and tetralogy of Fallot (TOF) [11]. However, the pathogenic mechanism remains unclear. A study [12] has shown that CITED2 affects the development of cardiac neural crest by binding to TFAP2 and p300/CBP. Cardiovascular malformations and abnormal development of left-right asymmetric structures in CITED2 mice may also be due to dysfunction of the Nodal-Pitx2c pathway [13]. As a negative regulator of hypoxia-inducible factor-1 (HIF-1), CITED2 plays an important role in cardiac development, and the coupling of myocardial and coronary artery growth in the heart may occur in part through the Cited2-Vegfa pathway [14]. Further, abnormal coronary vessels in CITED2 mice may cause continuous myocardial hypoxia, which further increases the activity of HIF1 and the expression of VEGF, thus forming a vicious circle, eventually leading to the occurrence of abnormality of the cardiac outflow tract and septal defects [15].

Promoters are located in the DNA regulatory regions upstream of genes and play an important role in transcriptional regulation [16]. Depending on the location and nature of the gene defect, variants in the promoter region of a gene may disrupt the orderly recruitment of TFs (Transcription factors) in the promoter region, thereby disrupting the normal process of gene activation. Therefore, promoter variants can decrease or increase mRNA levels and thus increase protein levels [17]. In our previous study of VSD patients, it was found that variants in promoter regions do significantly affect gene expression [18,19]. However, the role of the variants of CITED2 promoter on the development of ASD has not been reported. We hypothesized that variants of the CITED2 gene promoter may be involved in the formation of ASD. To test this hypothesis, in this study, we performed variant screening on the DNA sequence of the CITED2 gene promoter region in ASD patients in comparison to healthy controls. Further, the function of the variants was analyzed at the cellular level.

## 2. Materials and Methods

### 2.1. Participants

**Figure 1:**
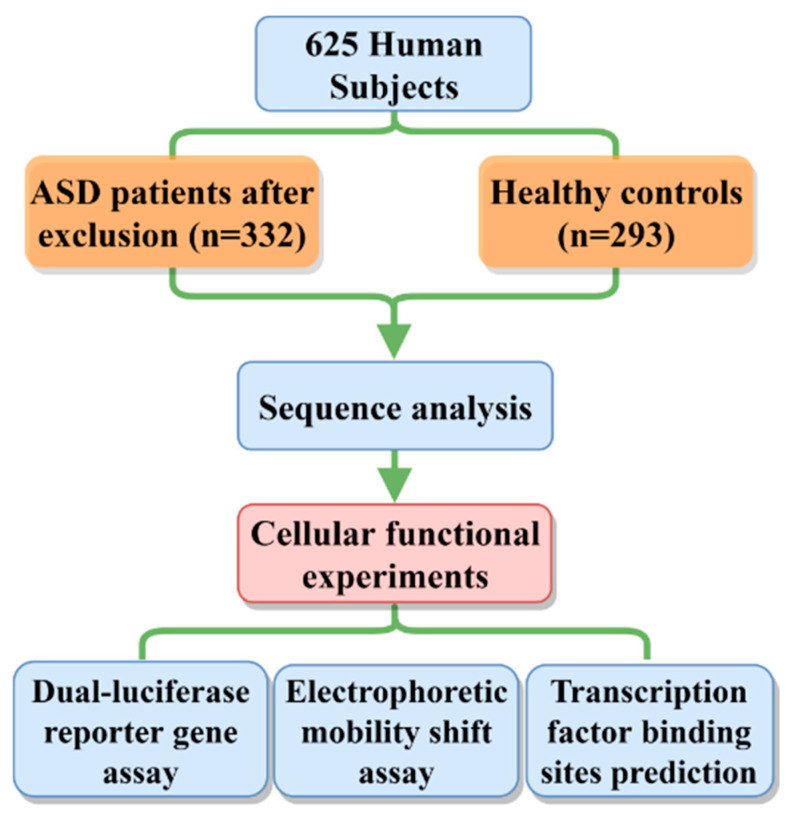
Study flow chart and experimental procedures. A total of 625 subjects participated in the study. Sequence analysis, cell function experiments, electrophoretic mobility shift analysis, and bioinformatics analysis were performed.

This study was approved by the Institutional Review Board of TEDA International Cardiovascular Hospital (clinical research ethics review No. 2021-0715-4) and followed the principles of the Declaration of Helsinki. Written informed consent was obtained from the parents or guardians of all subjects. Blood samples were collected from 625 subjects, including 332 isolated ASD patients and 293 healthy controls. All the patients had no known family history of CHDs or other genetic disorders. We defined the ASD from these patients as “Sporadic ASD”. All patients underwent corrective surgery at TEDA International Cardiovascular Hospital, Tianjin University, China. The patients and the control subjects did not differ significantly in terms of race, gender or age. The control group was selected from routine health examinations or CHD screening programs. All control subjects confirmed no family history of heart disease or other genetic disorders. Further, the control subjects had no cardiac defects or other major diseases based on body check and echocardiography (Figure 1).

### 2.2. Genomic DNA was Extracted and Subjected to Sequence Analysis

Genomic DNA was extracted from peripheral blood using a blood genomic DNA extraction kit (TIANGEN, China) following standard procedures. The primers were designed based on the reference sequence (NCBI: NG_016169.1) to cover the CITED2 promoter and flanking sequence, from −1197 to +220 to the transcription start point (+1). The 1418 bp segments were amplified by PCR, and the products were sequenced directly by Sanger sequencing as previously reported [18,19,20,21]. The primers required for PCR amplification and Sanger sequencing are shown in Table 1.

### 2.3. Cell Level Validation

#### 2.3.1. Plasmid Construction

The reporter plasmid pGL3 Basic-CITED2 promoter (WT) with firefly luciferase reporter is stored in our lab. Variants identified in the study were separately introduced into WT plasmid using a site-directed Gene Mutagenesis Kit (Beyotime, Nantong, China) according to the manufacturer’s protocol. The primer sequences used are shown in Table 1. All inserted variants were Sanger sequenced to ensure there were no variants other than the target variant.

#### 2.3.2. Cell Culture and Transfection

HEK-293 and HL-1cells were resuscitated in complete MEM/DMEM (10% fetal bovine serum +1% 100 units/mL penicillin and streptomycin). Approximate 2−6 × 10^5^ cells were cultured into a six-well plate one day before transfection (18–24 h). When cells were grown to 60–70% confluence, the reporter plasmids were transfected with the expressing renilla luciferase reporter plasmid (pRL-SV40) as an internal control to standardize transfection efficiency. The empty pGL3-basic vector was utilized as a negative control.

#### 2.3.3. Dual-Luciferase Reporter Gene Assay

Cells were collected and fully lysed after 48 h. Luciferase activities were measured using the specified dual luciferase reporter assay system (Thermo Scientific Fluoroskan FL, Waltham, MA, USA). The transcriptional activities of the CITED2 gene promoter were assessed by measuring the relative values of firefly luciferase activity and renilla luciferase activity. The experiment was independently repeated three times, each in triplicate.

To ensure the results form HEK-293 cells are repeatable, we also repeated similar experiments in the HL-1 cell.

#### 2.3.4. Transcription Factor Binding Site (TFBs) Prediction

The JASPAR database was used to further study the changes of transcription factor binding sites in the CITED2 promoter region with variants. The relative profile score threshold was set to 85%. JASPAR is an open-access database of annotated, high-quality, matrix-based transcription factor binding site profiles for multicellular eukaryotes [22]. Bioinformatics analysis using the JASPAR database according to Fornes and associates [23] indicated the disruption or creation of potential TF binding sites. The detailed procedures are listed in the JASPAR database (https://jaspar.genereg.net/, accessed on 1 February 2022). By comparison of the predicted results of VT and WT, the disruption (lost TFBs) or creation (new TFBs) of potential TF binding sites can be determined.

#### 2.3.5. Electrophoretic Mobility Shift Assay (EMSA)

EMSA assay was used to detect the effect of variants identified in the CITED2 promoter on potential transcription factors in the present study. Nuclear proteins were extracted from HEK-293 cells using a nuclear protein extraction kit (Beyotime, Nantong, China). The oligonucleotides (30 bp) were biotin-labelled as probes. The biotinylated double-stranded oligonucleotides with or without a variant were synthesized and used as the labeled probe. The results of gel imaging were obtained according to the Chemiluminescent EMSA Kit (Beyotime, Nantong, China).

### 2.4. Statistical Analysis

Statistical analysis was performed using IBM SPSS 23.0 software. Continuous variable data were expressed as mean ± standard error (SEM), and quantitative data were compared using standard Student’s *t*-test. Pearson’s chi-square test was used to compare the variant frequency of the CITED2 gene promoter DNA sequence between the experimental group and the control group. *p* < 0.05 was considered significant difference.

## 3. Results

### 3.1. DNA Sequence Variants in ASD Patients and Healthy Controls

**Figure 2:**
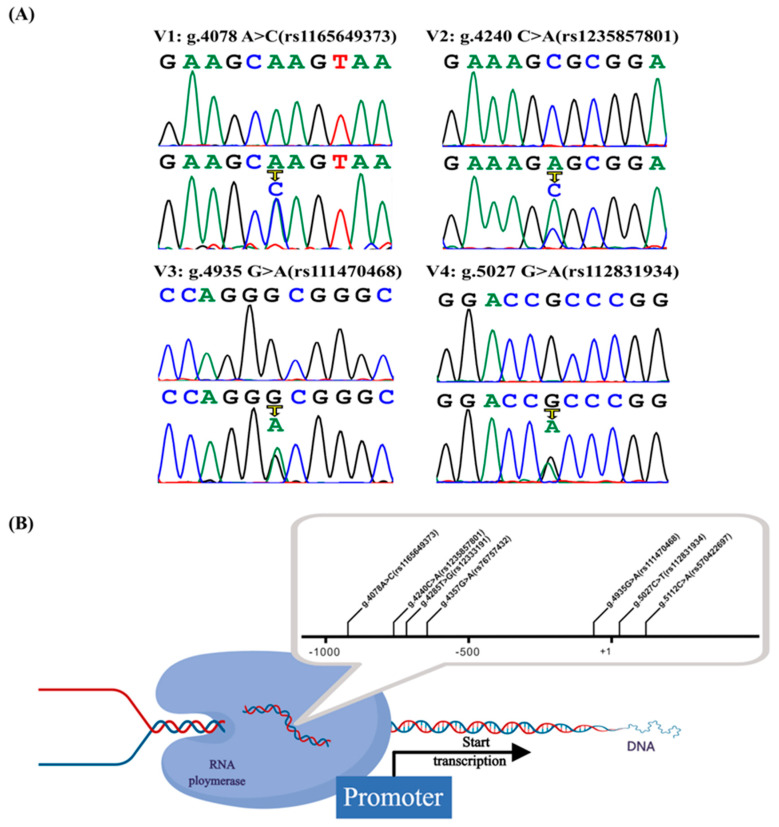
Mapping and Sanger sequencing map of CITED2 gene promoter variants. (**A**) The variants (V1–V4) of the CITED2 gene promoter found only in patients with atrial septal defect (ASD) are shown (g.4078A>C(rs1165649373), g.4240C>A(rs1235857801), g.4935C>T(rs111470468), g.5027C> T (rs112831934)). The top panel of V1-V4 shows the wild type and the bottom panel shows the heterozygous variant. (**B**) Regulatory variants of the CITED2 gene. The transcription start point is located at position 5001 of the first exon (+1), and these genetic variants are named according to the genomic sequence of the human CITED2 gene (Genbank Accession No.NG_016169.1), describing the location of the CITED2 gene promoter variants.

DNA sequence was performed in the 625 subjects (332 ASD patients and 293 healthy controls). Seven variants were found. Among these seven variants, there were four variants (g.4078A>C(rs1165649373), g.4240C>A(rs1235857801), g.4935C>T(rs111470468), g.5027C>T(rs112831934)) that were only found in the ASD patients with zero incidence in the controls (Figure 2). Importantly, the variant g.4935C>T(rs111470468) was found in four ASD cases, and the variant g.5027C>T(rs112831934) was found in two ASD cases. In the GnomAD database (http://gnomad-sg.org/, accessed on 2 February 2022) and ALFA database (https://www.ncbi.nlm.nih.gov/snp/, accessed on 2 February 2022), g.4078A>C(rs1165649373), g.4240C>A(rs1235857801), g.5027C>T(rs112831934) total allele frequency < 0.1%. G.4935C>T(rs111470468) is quite common in other populations, with MAF > 0.02 in the European population, but MAF = 0.000 in the East Asian population. These four variants were further validated at the cellular level. There were two common single nucleotide polymorphisms (SNPs) found in the normal control (g.4285T>G(rs12333191) in 33 cases and g.4357G>A(rs76757432) in 16 cases) and in the ASD patients (16 and 3 patients, respectively). Further, one variant [g.5122C>A(rs570422697)] was only found in the control group. The above three variants were excluded from further studies (Table 2).

### 3.2. Functional Analysis of Variants with Dual-Luciferase Reporter Genes

**Figure 3:**
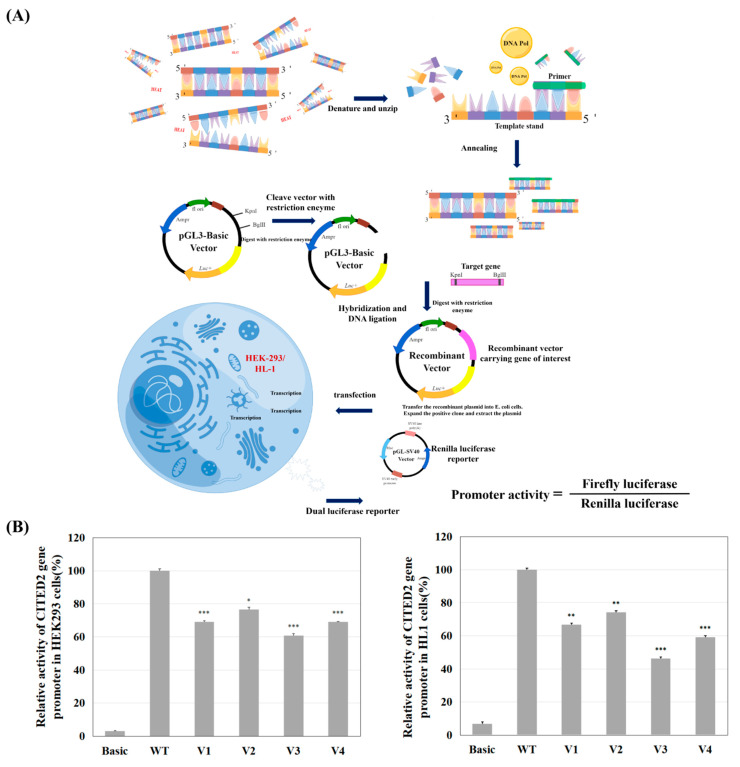

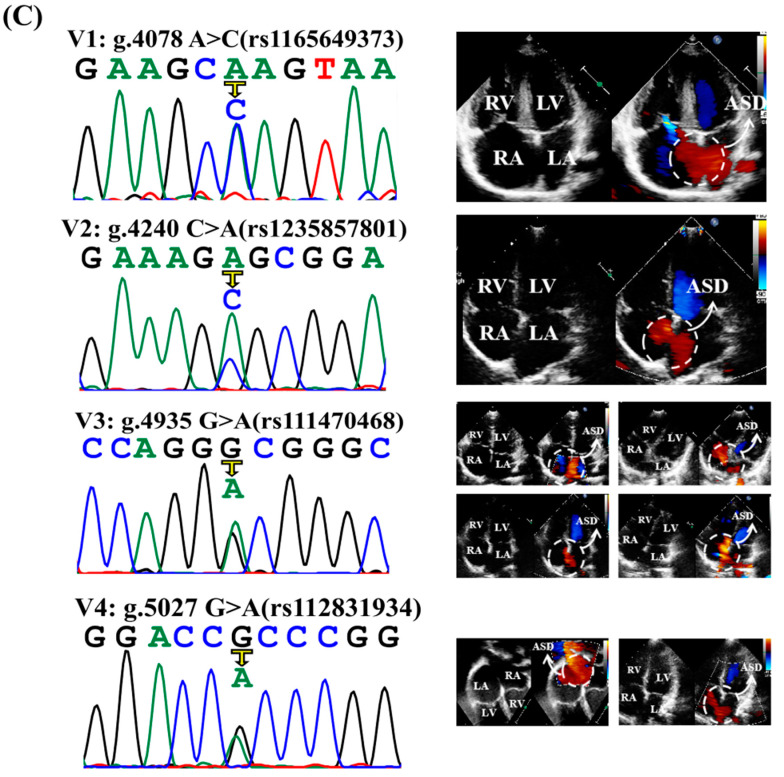
Dual-luciferase reporter gene assay process and echocardiography of ASD patients. (**A**) Procedure of cell function experiments. To confirm the effect of variants on promoter activity, wild-type and variant CITED2 gene promoter fragments were prepared, gene expression vectors were constructed, and HEK-293 cells and HL-1 cells were transfected. The transcriptional activity of wild-type and variant CITED2 gene promoters was detected by dual-luciferase reporter assay. DNA Pol, DNA polymerase. (**B**) Relative transcriptional activity of the CITED2 gene promoter in HEK293 (left panel) and HL-1 cells (right panel). The transcriptional activity of the wild-type CITED2 gene promoter was set as 100%, and the relative activity of the variant was calculated. Results are presented as the mean ± SD of three independent experiments, each in triplicate. pGL3-Basic: empty vector; WT: wild type; V1: pGL3-4078A>C, V2: pGL3-4240C>A, V3: pGL3-4935C>T, V4: pGL3-5027C>T. *, *p* < 0.05; **, *p* < 0.01; ***, *p* < 0.001. (**C**) Color Doppler echocardiography of parasternal short-axis view at aortic valve level. The imagine shows the four cardiac chambers (left Echo panels) and the left-to-right blood flow shunt from the left atrium to the right atrium through the ASD (dotted line circle and the arrow) shows ASD (arrow) in patients with variants of g.4078A>C, g.4240C>A, g.4935C>T, g.5027C>T. LV, left ventricle; LA, left atrium; RA, right atrium; RV, right ventricle; ASD, atrial septal defect.

To further determine whether these variants of the CITED2 gene promoter directly affect the activity of the CITED2 promoter, we constructed different reporter plasmids based on the CITED2 promoter fragment: empty pGL3 (negative control), pGL3-WT (wild-type CITED2 gene promoter), pGL3-4078C, pGL3-4240A, pGL3-4935T, and pGL3-5027T. These reporter gene assays were performed after the transfection of HEK-293 cells. The experimental process is shown in Figure 3A. As shown in Figure 3B left panel, luciferase activity analysis showed that among the four genetic variants tested for promoter activity, luciferase expression was significantly reduced compared to the wild type (*p* < 0.05). Furthermore, Figure 3B right panel shows that the results in HL-1 cells were well in accordance with that in HEK-293 cells. In Figure 3C, color Doppler echocardiograms in parasternal short-axis views at the level of the aortic valve for these four variants (eight patients in total) are shown.

### 3.3. Subsection Potential Binding Sites for TFs Are Affected by Genetic Variant

**Figure 4:**
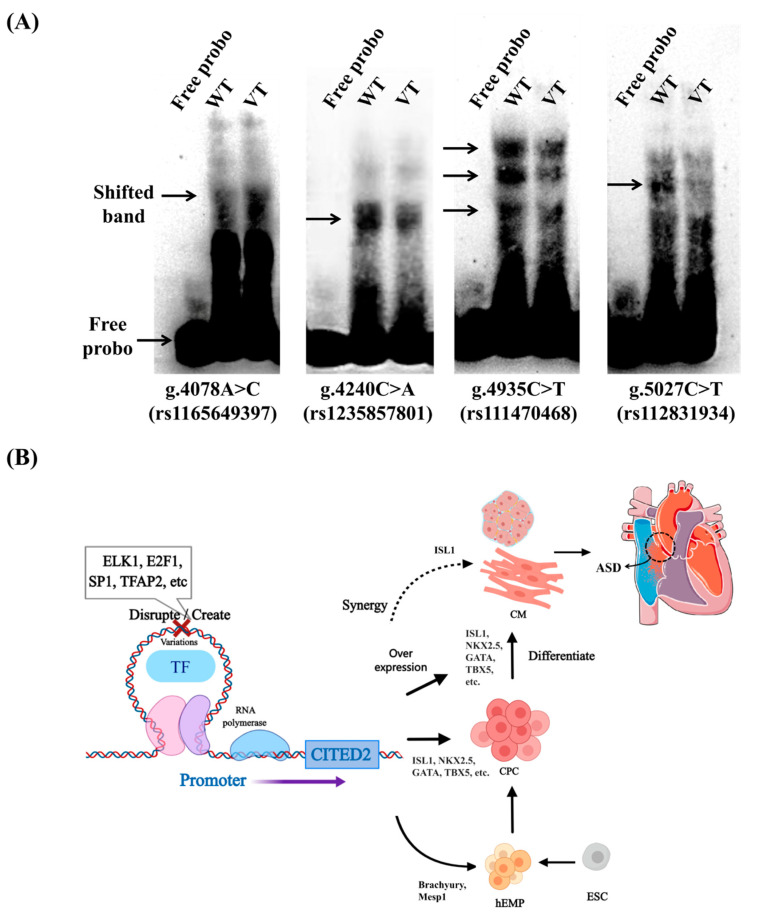
Electrophoretic mobility shift analysis (EMSA) experiments and schema for effect of variants in the CITED2 promoter region on possible pathways in cardiac development. (**A**) Results of EMSA of CITED2 gene promoter variants. The results show that the variants (g.4078A>C(rs1165649373), g.4240C>A(rs1235857801), g.4935C>T(rs111470468), g.5027C>T(rs112831934)) significantly affected transcription factor binding capacity. Free probes are marked on the bottom; arrows indicate affected binding of unknown transcription factors; WT, wild type; VT, variant. (**B**) Effect of variants in the CITED2 promoter region on some transcription factors and the possible consequence in cardiac development. CM, mature cardiomyocytes; CPC, Cardiac progenitor cells; hEMP, mesoderm progenitor cells; ESC, embryonic stem cells.

EMSA was performed with biotin-labeled oligonucleotides and HEK293 nuclear protein. The EMSA results are shown in Figure 4A. The results show that the variants (g.4078A>C(rs1165649373), g.4240C>A(rs1235857801), g.4935C>T(rs111470468), g.5027C>T(rs112831934)) significantly affected transcription factor binding capacity.

JASPAR is an open-access database of annotated, high-quality, matrix-based transcription factor binding site profiles for multicellular eukaryotes [22]. Bioinformatics analysis using the JASPAR database according to [23] indicated that all four variants may disrupt or generate potential TF binding sites.

Table 3 summarizes the analysis data. The E2F, HOX, NIF, ZNF and other families are covered in the table, among which ELK1, E2F1, SP1, TFAP2, etc. have been confirmed as transcription factors expressed by CITED2 [24,25,26,27]. Figure 4B establishes a graph of the effects of variants in the CITED2 promoter region on some transcription factors and their effects on cardiac development based on the validation at the cellular function level of this study, JASPAR database predictions, and previous findings.

## 4. Discussion

This study, for the first time, found in isolated ASD patients that (1) there are four variants (4078A, 4240A, 4953T, and 5027T) in the CITED2 promoter region that had zero frequency in the control cases in this study with low allele frequency (<0.001) in the total population or in the East Asian records from the GonomAD or the ALFA database; (2) these variants affect TFBs and significantly reduce CITED2 promoter activity, resulting in altered cellular function; and (3) the functional alterations caused by these variants may affect a set of downstream genes and pathways, ultimately leading to ASD.

The role of the variants in the coding region of CITED2 has been well described, but the variants in the promoter region of this gene have not been reported [11,27] as mentioned above. This study, for the first time, investigates the variants of the promoter region of the CITED2 gene in ASD patients and demonstrates the causing effect of these variants in the development of ASD.

To demonstrate that the variants are functionally meaningful at the cellular level, we validated them in two types of cells, HEK-293 and HL-1. In both experiments, it was shown that the variants (V4078, V4240, V4935, V5078) significantly altered CITED2 promoter activity and thus altered cellular function that is most likely related to altered TFBs. The EMSA results also show that the binding capacity of the transcription factors to the CITED2 promoter was altered by the increased/decreased brightness of the bands in the variants compared to the wild type (Figure 4A). TFs recognize specific DNA sequences to control chromatin and transcription, forming a complex system that directs genome expression [28]. TFs regulate the expression of specific genes, so that heart-specific cells differentiate and develop into the heart. Variants in these transcription factors and the genes they regulate can lead to CHD. CITED2 is an important member of the CITED family of transcriptional co-activators and plays a key role in the regulation of expression and in the early and late stages of cardiac development [29]. However, a single transcription factor cannot completely control the growth and development of the heart. Specific combinations of cardiac-specific and ubiquitously expressed transcription factors are required to activate some cardiac genes in transfected cells, resulting in distinct gene expression states that reflect distinct physiological processes [17]. ASD is by far the most studied type of isolated CHD [30]. Cardiac-specific transcription factors (GATA4 and NKX2-5), extracellular signaling molecules (VEGFA and BMP10), and cardiac proteins (MYL2, MYL3, MYH7, TNNT1, and TNNT3) are down-regulated in ASD patients, which may affect cardiac atrial septum formation, cardiomyocyte proliferation and myocardial development [27,31]. In some reports, CITED2 variants in the coding region were detected to be associated with cardiac malformations, including ASD, VSD, TOF, etc. [11,32]. TFs, two chromatin-modifying enzymes, and TFs combine to activate genes and are recruited to promoters in precise order. However, timing or failure to recruit transcription factors may affect the transcriptional regulation of genes, leading to disease [33].

Combined with the predicted results of the JASPAR database and previous studies, it is shown that the CITED2 promoter variant leads to the reduction in CITED2 activity, which may be directly involved in the occurrence and development of ASD. The reason for the reduced gene activity may be the overexpression of some heart-related genes associated with the low expression of CITED2, such as HIF1α and VEGA [24]. In addition, its low expression also reduces the activity of related pathways such as Nodal, Lefty2, Pitx2, TFAP2 [25], and other pathways and hinders the normal development of the heart. CITED2 is required for normal expression of mesoderm markers such as Brachyury and Mesp1 and cardiac mesoderm markers such as Isl1, NKX2.5, Gata4, and Tbx5. In embryotic stem cells, overexpression of CITED2 triggered increased expression of Isl1, NKX2.5, Gata4, and Tbx5, which favored cardiac differentiation [32]. In fact, CITED2 and Isl1 proteins physically interact and synergistically promote cardiac differentiation [34].

Interestingly, the variant g.4078A > C (rs1165649373) found in an ASD patient in the present study was also found in the VSD patients from our previous study [18]. This fact suggests that this variant is an important pathogenic cause for not only ASD, but also other CHD as often seen for other genes.

This study has certain limitations. The interaction between the discovered variants at the CITED2 gene promoter region and the downstream genes needs to be further validated. In addition, the biological effect of these variants needs to be validated in the in vivo animal model. These considerations will be taken into account in our future investigation.

## 5. Conclusions

In conclusion, this study is the first to identify genetic variants in the promoter region of the CITED2 gene in patients with ASD in the Chinese Han population. Furthermore, in cellular function experiments, these variants are shown to indeed significantly affect the expression of the CITED2 gene. Bioinformatics analysis also indicates that these variants are involved in the development of ASD. Therefore, this study provides new insights into the etiology and potential treatment strategies of CHD.

## Figures and Tables

**Table 1:** Primers used in this study.

Primers Name	Sequences 5’–3’	Location	Position
PCR and sequencing primers			
CITED2-F1	5’-AAAGGAAGAGTCCCAGCCGT-3’	3804	−1197
CITED2-F2	5’-TTTCTGCTCCGAAGACCGAG-3’	5221	+200
Primers containing restriction sites			
CITED2-KpnI ^a,b^	5’-(KpnI)-**GG**GGTACCAAAGGAAGAGTCCCAGCCGT-3’	3804	−1197
CCCAGCCGT-3’			
CITED3-BglII ^a,b^	5’-(BglII)-**CA**AGATCTTTTCTGCTCCGAAGACCGAG-3’	5221	+200
AGACCGAG-3’			

Note: PCR primers are designed based on the genomic DNA sequence of the CITED2 gene (NG_016169.1). The transcription start site is at the position of 5001 (+1). Abbreviations: F, upstream primer; R, downstream primer. ^a^ Limit position underscore. ^b^ Protective bases are shown in bold.

**Table 2:** Variants found in the CITED2 gene promoter region in patients with atrial septal defect in comparison with healthy controls.

Variations	ASD ^a^	Controls ^a^	Position ^b^	Genotypes	Allele Frequency ^c^		*p*-Value
**Frequency in Control = 0 (Further Validation)**					**Total**	**East Asian**	
g.4078A>C (rs1165649373)	1	0	−923 bp	AC	G = 0.00003185	G = 0.0006410	>0.9999 ^#^
g.4240C>A (rs1235857801)	1	0	−761 bp	CA	T = 0.00000 *	T = 0.000 *	>0.9999 ^#^
g.4935C>T (rs111470468)	4	0	−66 bp	CT	A = 0.01546	A= 0.000	0.127 ^#^
g.5027C>T (rs112831934)	2	0	+26 bp	CT	A = 0.0005414	A = 0.003205	0.501 ^#^
**Frequency in Control ≠ 0 (No Further Validation)**							
g.4285T>G (rs12333191)	16	33	−716 bp	TG	C = 0.231281	C = 0.0467	0.00278
g.4357G>A (rs76757432)	3	16	−644 bp	GA	T = 0.094422	T = 0.0006	0.001^#^
g.5122C>A (rs570422697)	0	1	+121 bp	CA	T = 0.000029	T = 0.0013	0.469 ^#^

Abbreviations: −, not applicable; ASD, atrial septal defects. ^a^ Allele frequency in groups. ^b^ Variations are located upstream (−) to the transcription start site at the position of 5001 (+1) of CITED2 gene (NG_016169.1). ^c^ The allele frequency was obtained from the GnomAD database. ^*^ Allele frequency not found in the GonomAD database but shown in the ALFA database (version: 20201027095038). *p*-value was calculated by Chi-square or Fisher’s exact test (^#^).

**Table 3:** Effects of the promoter region variants of the CITED2 gene on TFBS predicted by JASPAR database.

Variations	Binding Sites for Transcription Factors		Promoter Activity
	Create	Disrupt	
g.4078A>C (rs1165649373)	NFIX, BHLHA15, RHOXF1, NFIC, ELK::HOXA3, ASCL1	HOXB7, ZNF714, HOXC8, ISL2	**↓**
g.4240C>A (rs1235857801)	ETS1	-	**↓**
g.4935C>T (rs111470468)	ZNF354C, MAZ, EHF, ETV4	E2F1, E2F6, EBF1, KLF1, KLF14, KLF2, KLF5, KLF6, NR2C2, SP1, SP2, SP4, TFAP2B, ZNF704	**↓**
g.5027C>T (rs112831934)	ZNF354C, ERF::NHLH1, ZNF273, NR2C2, YBX1	RFX5, THAP1, ZNF704	**↓**

Abbreviations: -, not applicable; TFBS, transcription factor binding sites.

## Data Availability

The individual SNP numbers are given in Table 2. The genetic variants described in this manuscript are available at https://www.ncbi.nlm.nih.gov/snp/, accessed on 2 February 2022. Data supporting the findings of this study are available upon reasonable request from the corresponding authors.
